# Continuum of Core 1 biomarkers in preclinical Alzheimer’s disease

**DOI:** 10.1186/s13195-026-02044-1

**Published:** 2026-04-10

**Authors:** Leonardino A. Digma, Christina B. Young, Joseph R. Winer, Karly A. Cody, Kyan Younes, Jintao Sheng, Philip S. Insel, Robert A. Rissman, Reisa Sperling, Elizabeth C. Mormino

**Affiliations:** 1 Department of Neurology and Neurological Sciences, Stanford University School of Medicine, 3145 Porter Drive Palo Alto, Stanford, CA 94304 USA; 2 Department of Psychology, Stanford University, Stanford, CA USA; 3 Wu Tsai Neurosciences Institute, Stanford University School of Medicine, Stanford, CA USA; 4 Department of Psychiatry and Behavioral Sciences, University of California San Francisco, San Francisco, CA USA; 5 Alzheimer’s Therapeutic Research Institute, Keck School of Medicine, University of Southern California, San Diego, CA USA; 6 Brigham and Women’s Hospital, Harvard Medical School, Boston, MA USA; 7 Molecular Imaging Program at Stanford, Stanford University, Stanford, CA USA

**Keywords:** Biological staging, preclinical Alzheimer’s disease, p-tau217, tau-PET

## Abstract

**Background:**

Biological Staging for Alzheimer’s disease (AD) in clinically unimpaired (CU) individuals is critical for early detection efforts. In this study, we evaluated whether Core 1 biomarkers (plasma p-tau217 and amyloid-PET) within Biological Stage A, the earliest biological stage of AD, predict progression of downstream biomarkers and cognition.

**Methods:**

We used baseline plasma p-tau217 and amyloid-PET, and longitudinal tau-PET, atrophy, and cognition data from the recently completed Anti-Amyloid Treatment in Asymptomatic Alzheimer’s (A4) Study. PET data were used to identify participants within AD Biological Stage A (amyloid-PET positive and medial temporal tau-PET negative). Within these Stage A participants, linear mixed effects models were used to examine associations between baseline levels of plasma p-tau217 and amyloid-PET burden with longitudinal regional tau-PET, atrophy, and cognition. We additionally evaluated whether p-tau217 and amyloid-PET burden within this group were associated with higher risk of progression to Biological Stage B+ (tau-PET positive in the medial temporal lobe). In our statistical models, we included covariates for age, sex, and APOE4 carriage.

**Results:**

Of 335 A4 participants with complete biomarker data, 222 were identified as being in Biological Stage A. Among Biological Stage A CU, baseline plasma p-tau217 and amyloid-PET burden were associated with faster tau-PET accumulation and atrophy in AD-relevant regions (mean [SD] follow-up time for tau-PET: 4.2 [2.1] years and MRI: 4.2 [1.9] years), as well as faster cognitive decline (mean [SD] follow-up time for PACC: 5.7 [1.6] years) (all *p* < 0.05). Plasma p-tau217 and amyloid-PET burden were also associated with higher risk of progression to Biological Stage B+.

**Discussion:**

In CU individuals in the initial stage of AD (Biological Stage A), early changing AD biomarkers provide prognostic information of downstream markers of disease. Evaluation of the utility of these measures in a real-world setting is warranted.

**Trial registration:**

The A4 study was submitted for registration to clinicaltrials.gov on December 6th, 2013. The study is registered with ID NCT02008357. Screening and data collection for the study began in April 2014.

**Supplementary Information:**

The online version contains supplementary material available at 10.1186/s13195-026-02044-1.

## Background

Alzheimer’s disease (AD) is defined by the presence of amyloid plaques and tau tangles in the brain [1]. Advances in biomarker technologies have enabled the detection of these protein aggregations during life with measures from cerebrospinal fluid (CSF), positron emission tomography (PET) brain imaging, and, more recently, plasma. The ability to measure the pathological hallmarks of disease in vivo has motivated multiple biological frameworks and staging schemes for AD. An updated framework was recently published by a workgroup of stakeholders [2], proposing a sequence of four Biological Stages (A through D) along the AD continuum based on biomarker profiles.

The 2024 biological criteria does not provide a specific guidance on the operationalization of the proposed biological stages. Rather, it proposes a flexible schema that can be applied based on available biomarker information. Broadly, the criteria suggests a set of early changing “Core 1” biomarkers that, if positive, places a patient on the AD continuum (i.e., at least Biological stage A). Biomarkers deemed appropriate for Core 1 include measures of amyloid (A, amyloid-PET, CSF or plasma measures of Aβ42) and early changing measures of tau (T_1_, CSF or plasma measures of p-tau181, 217, or 231). Individuals with a positive Core 1 biomarker can be further staged with “Core 2” biomarkers of disease prognostication, such as tau-PET (T_2_). Elevated tau-PET signal in the medial temporal lobe (MTL) is proposed to define Biological Stage B, whereas moderate and high levels of neocortical tau-PET are recommended for Stage C and Stage D, respectively. Currently, tau-PET is the only recommended Core 2 biomarker that has sufficient data supporting its utility in the biological framework as a marker of disease progression. Newer T_2_ biomarkers, such as MTBR-tau243 [3], are noted for their initial promise but warrant further validation. Using this schema and available biomarkers, several groups have recently applied the proposed framework in observational studies of aging and AD [4–8].

As noted above, the earliest biological stage in the AD continuum is Biological Stage A and an individual can meet criteria for this stage by having an abnormal Core 1 biomarker (e.g., elevated amyloid-PET or plasma p-tau). An open question, however, is whether these early changing Core 1 biomarkers provide additional information beyond just binary statuses during the earliest stages of disease. In other words, when restricted to amyloid + yet tau-PET negative participants (Biological Stage A), do continuous levels of Core 1 biomarkers predict subsequent changes relevant for disease progression. While biomarker measures of amyloid tend to show a bimodal distribution, there is evidence that meaningful variability exists beyond binary positive and negative status in cognitively unimpaired (CU) individuals [9, 10]. Although plasma ptau-217 is a newer marker, there is also data demonstrating that variability in this measure can reveal groups at higher risk for disease progression [11, 12]. Studies examining the continuum of early changing Core 1 biomarkers [10–14], however, do not exclude tau-PET+ individuals, and it is possible that reported effects of plasma p-tau217 and/or amyloid-PET burden are driven by the inclusion of this subgroup that is further along the AD trajectory (i.e., Stage B or higher).

We therefore sought to determine whether continuous values across two established Core 1 biomarkers—amyloid-PET and plasma p-tau217—were associated with subsequent disease relevant changes among Biological Stage A, CU individuals. Although these markers are classified as Core 1 in the new framework and are shown to correlate strongly across the AD spectrum [15–17], they nevertheless measure distinct aspects of disease biology, remain dynamic in the earliest stages of AD, and thus may provide unique information during the earliest stage of AD. To evaluate the prognostic value of these early markers we applied the new staging criteria in a large sample of CU older adults, and determined whether the continuum of Core 1 biomarkers was predictive of subsequent longitudinal change in downstream disease markers within Biological Stage A.

## Methods

### Study participants

**Fig. 1:**
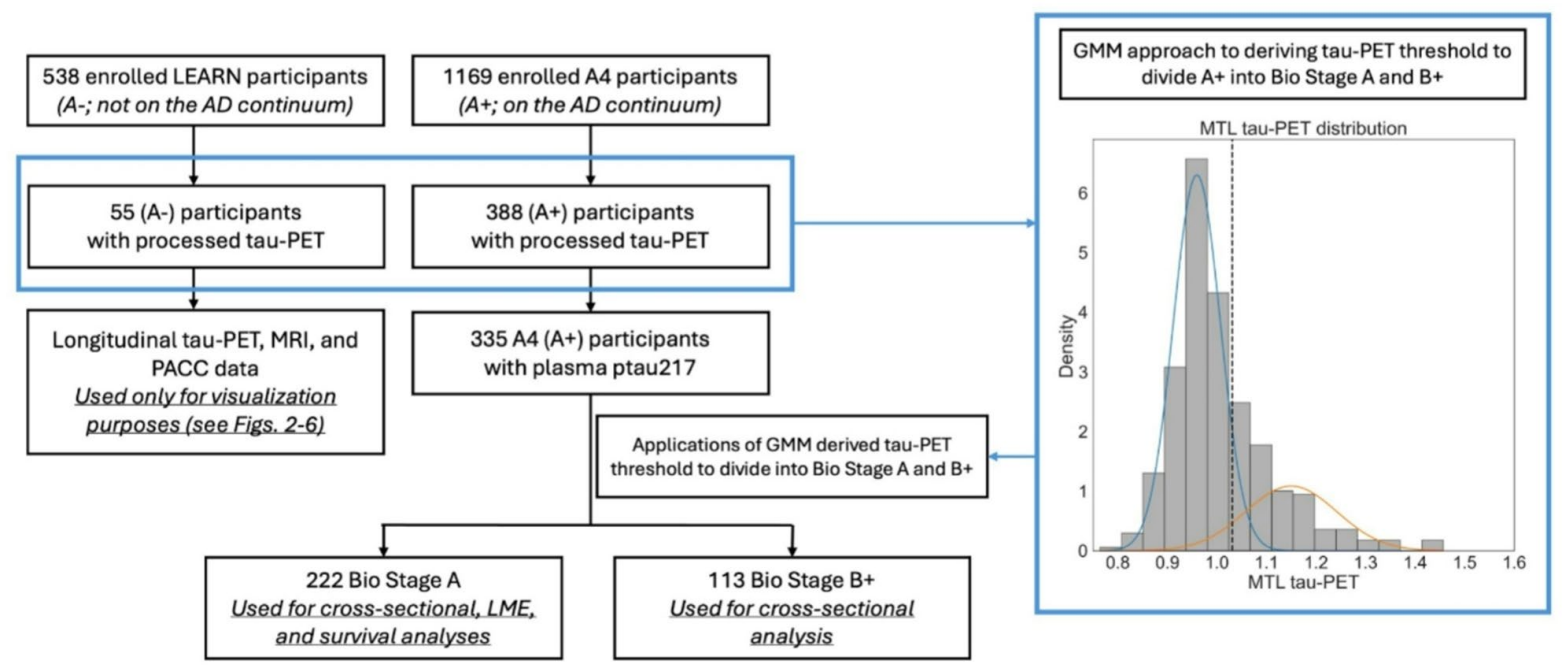
Flow chart illustrating selection of participants for analysis. From the A4 study, we included patients with complete amyloid-PET, tau-PET, and p-tau217 data. From the LEARN study, we included participants that had tau-PET data. Gaussian mixture modeling was applied to the tau-PET data from A4 and LEARN to derive an MTL tau-PET threshold. This threshold was applied to the A4 participants to derive Biological Stage A and Biological Stage B+ groups

Participant demographic, biomarker, and neuropsychological data were downloaded on November 5th, 2024 (a4studydata.org). We used data from participants that were enrolled in the A4 clinical trial (variable *SUBSTUDY*, value ‘A4’) and had amyloid-PET, plasma p-tau217, tau-PET, and MRI data available (Fig. 1). The inclusion and exclusion criteria for the A4 trial have been described in detail previously [18]. Briefly, patients with normal cognition and evidence of amyloid in the brain, by a combination of visual and quantitative measurements, were enrolled in A4 and then randomized to either solanezumab or placebo. A subset of participants that were not eligible for A4 due to not having elevated brain amyloid (and thus not on the AD spectrum) were enrolled in the LEARN study. The rationale for the LEARN study was to improve the general understanding of longitudinal change in amyloid negative CU participants undergoing the same study protocol as amyloid positive participants that continued into the A4 trial (albeit in the absence of randomization into treatment and placebo arms) [19]. For our analyses (Fig. 1), LEARN participants (variable *SUBSTUDY*, value ‘LEARN’) that underwent tau-PET imaging were included for determining tau-PET positivity threshold (see below). Data from LEARN was not included in any of the other cross-sectional or longitudinal statistical analyses since the focus of this work was on Amyloid+ participants. Lastly, LEARN (Amyloid-) participants were included for visualization purposes in some Figures, to contextualize patterns of change observed in the Amyloid+ groups.

### Biomarker and cognitive measures

We used florbetapir-positron emission tomography (amyloid-PET), flortaucipir-PET (tau-PET), and T1-weighted magnetic resonance imaging (MRI) data. For amyloid-PET, a summary standard uptake value ratio (SUVr) was calculated for each participant using previously described methods [20, 21] (variable *Composite_Summary* from the spreadsheet *imaging_SUVR_amyloid.csv*), and converted to centiloids [22]. The *Composite_Summary* averages amyloid-PET signal from frontal, temporal, parietal, and cingulate regions, and the reference region for amyloid-PET was the whole cerebellum. For tau-PET, regional tau burden was estimated by calculating regional tau-PET SUVrs from a template atlas derived from participants from the Harvard Aging Brain Study [23] and normalized by a Parametric Estimation of Reference Signal Intensity (PERSI) derived reference region [24]. Briefly, the PERSI approach fits a bimodal Gaussian distribution to the histogram of signal intensities from the white matter. The mean and standard deviation from the first Gaussian are used to calculate the full width at half maximum (FWHM). White matter voxels with intensity values within the FWHM are then included in tau-PET the reference region. For our statistical analyses (see below) we used regional tau data from entorhinal, parahippocampal, fusiform, and inferior temporal. These data were extracted from *imaging_SUVR_tau.csv*. For MRI, the T1-weighted images were processed locally using the FreeSurfer longitudinal processing stream [25, 26]. For our analyses, we used regional thickness values from entorhinal, parahippocampal, fusiform, and inferior temporal.

We used baseline plasma p-tau217 levels (variable *ORRES*, spreadsheet *biomarker_p-tau217.csv*). Plasma p-tau217 levels were measured using an ECL immunoassay, performed on the Meso Scale Discovery Sector S Imager 600 MM, as previously described [27]. In the released data, some subjects had raw baseline plasma p-tau217 levels (variable *ORRESRAW*) that were below the limits of the assay’s quantification. These values were excluded for primary analyses, but included in a sensitivity analysis.

To examine cognition, we used the preclinical Alzheimer cognitive composite (PACC) (variable *PACC*, spreadsheet *PACC.csv) *[28].

### Identification of Biological Stage A participants

Amyloid positivity for inclusion into A4 was determined using a hybrid quantitative/qualitative approach implemented by the A4 study team. Participants with a summary florbetapir SUVr > = 1.15 (corresponding to a centiloid value of 33.3) were considered amyloid positive (amyloid+); participants with summary SUVr 1.10–1.15 (between 24.3 and 33.3 centiloids) were also considered amyloid + if two readers gave consensus positive reads. Only participants classified as amyloid+ using this approach were eligible to enroll into A4 and undergo treatment randomization. A subset of 500 amyloid- individuals that did not reach this amyloid+ criteria enrolled into LEARN [19].

We divided A4 (amyloid+) participants into Biological Stage A and Stage B or higher (Stage B+). Per the latest criteria, Stage B + can be defined by amyloid+ in conjunction with MTL tau positivity detected with PET. To define MTL tau-PET positivity, we calculated a weighted average SUVr from bilateral entorhinal and parahippocampal cortex (variables *entorhinal_lh*,* entorhinal_rh*,* parahippocampal_lh*,* parahippocampal_rh*, spreadsheet *imaging_SUVR_tau*.csv) (Supplemental Methods 1). Gaussian mixture modeling (GMM) was applied to the MTL SUVr tau-PET distribution for all participants with tau-PET, using baseline MTL SUVr data only (Fig. 1). The threshold was set at 1.5 standard deviations (SD) above the mean of the tau-negative component from the GMM (threshold = 1.03). Importantly, we used a conservative 1.5 SD threshold (compared to, for example, 2 or 2.5 SD) to ensure removal of MTL tau-PET positive cases from Biological Stage A. Amyloid+ participants with an MTL SUVr below and above this threshold were Biological Stage A and B+, respectively.

### Statistical analyses

We first performed cross-sectional analyses to evaluate baseline pairwise associations between centiloid, plasma p-tau217, and MTL tau-PET, using Spearman correlations. We performed these cross-sectional analyses within the entire amyloid+ sample (Biological Stage A and B+ combined) and then within the Biological Stage A group.

Next, in our primary analyses, we ran a series of linear mixed effects (LME) models to examine whether baseline Core 1 biomarkers (plasma p-tau217 or centiloid) were associated with longitudinal change in downstream disease markers within Biological Stage A. Briefly, in each LME model, the outcome was either regional tau SUVr, regional thickness, or PACC score. For each model, the predictor variable of interest was p-tau217 and its interaction with time (Model 1a) or centiloid and its interaction with time (Model 1b). The models can be represented as:

$$\left(Model\;1a\right)Outcome\sim ptau217\ast time+covariates\ast time+\left(1+time\vert particiant\right)$$

$$\left(Model\;1b\right)Outcome\sim centiloid\ast time+covariates\ast time+\left(1+time\vert participant\right)$$

where *Outcome* is regional tau SUVrs (entorhinal, parahippocampal, fusiform, or inferior temporal), regional thickness (entorhinal, parahippocampal, fusiform, or inferior temporal), or PACC score. *Covariates* include age, sex, and APOE4 carriage. *time* denotes the time since the first tau-PET, MRI, or PACC date (see Supplemental Methods 2 for full details of derivation of the time variable). For the LME analysis in which PACC was the outcome, an additional covariate for education was also included. For the LME analysis in which regional thickness was the outcome, an additional co-variate for intracranial volume (ICV) was included; for visualization of these regional thickness analyses in Fig. 3, the regional thickness measures are shown as the residual after regression onto ICV. The (1 + *time | participant*) term indicates that random slopes and intercepts were included in all models.

Then, we examined whether the Core 1 biomarkers were independently associated with longitudinal change in downstream markers:

$$\left(Model\;1c\right)Outcome\sim ptau217\ast time+centiloid\ast time+covariates\ast time+\left(1+time\vert participant\right)$$

Estimates with *p*-value < 0.05 were considered significant. The temporal lobe regions used in the LMEs were selected because of the predilection of tau and atrophy for these regions early in the disease course; further, they were assessed separately in the LMEs, rather than as a composite, to explore whether the effects of Core 1 markers on tau and atrophy may differ by regions involved very early (i.e., entorhinal and parahippocampal) versus early (i.e., fusiform and inferior temporal) in AD. To illustrate the findings from the LME analyses, we used a median split to divide participants into those with high and low p-tau217 or centiloid values, and then plotted the trajectories for these groups. Dichotomization of plasma p-tau217 and centiloid was done for visualization purposes only (for all the linear mixed models, p-tau217 and centiloid were treated as continuous variables). The duration of the A4 study was about 4.5 years and, thus, most of the outcome measures were obtained within this time frame. Additional data points for outcome measures beyond 4.5 years were also collected during the open label extension period for the A4 study.

Lastly, we used survival analyses to evaluate progression to Biological Stage B+ within our Biological Stage A sample. Cox proportional hazards modeling was used to assess the effect of p-tau217 or centiloid values (treated as continuous variables) on the risk of progression to Biological Stage B+. P-tau217 values were z-scored so that hazard ratios can be interpreted as the risk associated with a 1 SD increase in p-tau217. Centiloid values were divided by 10 so that hazard ratios can be interpreted as risk associated with 10-point centiloid increase. An event was defined as a participant having a follow up tau-PET where their average MTL SUVr was above the threshold derived from the GMM procedure above. Following the same format as the LMEs (see above), we first evaluated p-tau217 and centiloid independently in separate models:

$$\left(Model\;2a\right)Surv\left(Time,Event\right)\sim ptau217+covariates$$

$$\left(Model\;2b\right)Surv\left(Time,Event\right)\sim centiloid+covariates$$

and then together in the same model to evaluate if they remained significant predictors of progression to Biological Stage B:

$$\left(Model\;2c\right)Surv\left(Time,Event\right)\sim ptau217+centiloid+covariates$$

Lastly, Kaplan Meier plots were used to illustrate survival probabilities for participants based on p-tau217 or centiloid level. For Kaplan Meier plots, participants were divided into high and low p-tau217 groups as well as high and low centiloid groups using a median split.

## Results

### Participant characteristics and baseline associations

There were 55 amyloid- (LEARN) participants with tau-PET data available and 335 amyloid+ (A4) participants with complete biomarker data (Fig. 1). Among the amyloid+ group, 222 (66%) were classified as Biological Stage A and 113 (34%) as Biological Stage B+. Compared to Biological Stage A participants, Biological Stage B+ participants had higher plasma p-tau217 (t=-6.55, *p* < 0.001) and centiloids (t=-3.81, *p* < 0.001)(Supplemental Result 1). There were no statistically significant differences in age, sex, education, APOE genotype, race, or drug group assignment between Biological Stage A and B+ participant groups (Table 1).

**Table 1:** Subject demographics. Demographics for the A4 participants whose data were used in this study. Data are represented as mean (standard deviation) unless otherwise noted. T-tests were used to compare continuous variables across groups and Chi-squared tests were used to compare categorical variables across groups. Abbreviations: MTL=medial temporal lobe, PET=positron emission tomography, SUVr=standardized uptake value ratio, MRI=magnetic resonance imaging, PACC=preclinical Alzheimer’s cognitive composite

	Biological Stage A	Biological Stage B+	
*N﻿*	222	113	
Age (years)	71.9 (4.8)	72.7 (4.8)	t=-1.43, *p* = 0.16
Sex (% female)	55.0%	61.9%	X = 1.22, *p* = 0.27
Education (years)	16.1 (3.0)	16.4 (2.7)	t=-0.81, *p* = 0.42
APOE4 (% carriers)	56.8%	61.1%	X = 0.41 *p* = 0.52
e2/e2	1	0	X = 5.35, *p* = 0.37
e2/e3	8	6	
e2/e4	8	2	
e3/e3	87	38	
e3/e4	106	55	
e4/e4	12	12	
Race			
White	205	99	X = 2.30 *p* = 0.68
Asian	9	8	
Black	5	3	
More than one race	2	2	
Unknown	2	1	
Treatment Group (% drug)	47.7%	54.0%	X = 0.93 *p* = 0.34
MTL tau-PET (SUVr)	0.96 (0.04)	1.13 (0.09)	t=-23.5, *p* < 0.001
Plasma p-tau217	0.25 (0.12)	0.36 (0.18)	t=-6.55, *p* < 0.001
Centiloid	61.2 (28.4)	74.7 (34.6)	t=-3.81, *p* < 0.001
tau-PET follow up time (years)	4.2 (2.1)	–	
tau-PET scans (# of scans per participants)	3.5 (1.3)	–	
MRI follow up time (years)	4.2 (1.9)	–	
MRI scans (# of scans per participants)	2.3 (0.77)	–	
PACC follow up time	5.7 (1.6)	–	
PACC administrations	11.6 (3.1)	–	

**Fig. 2:**
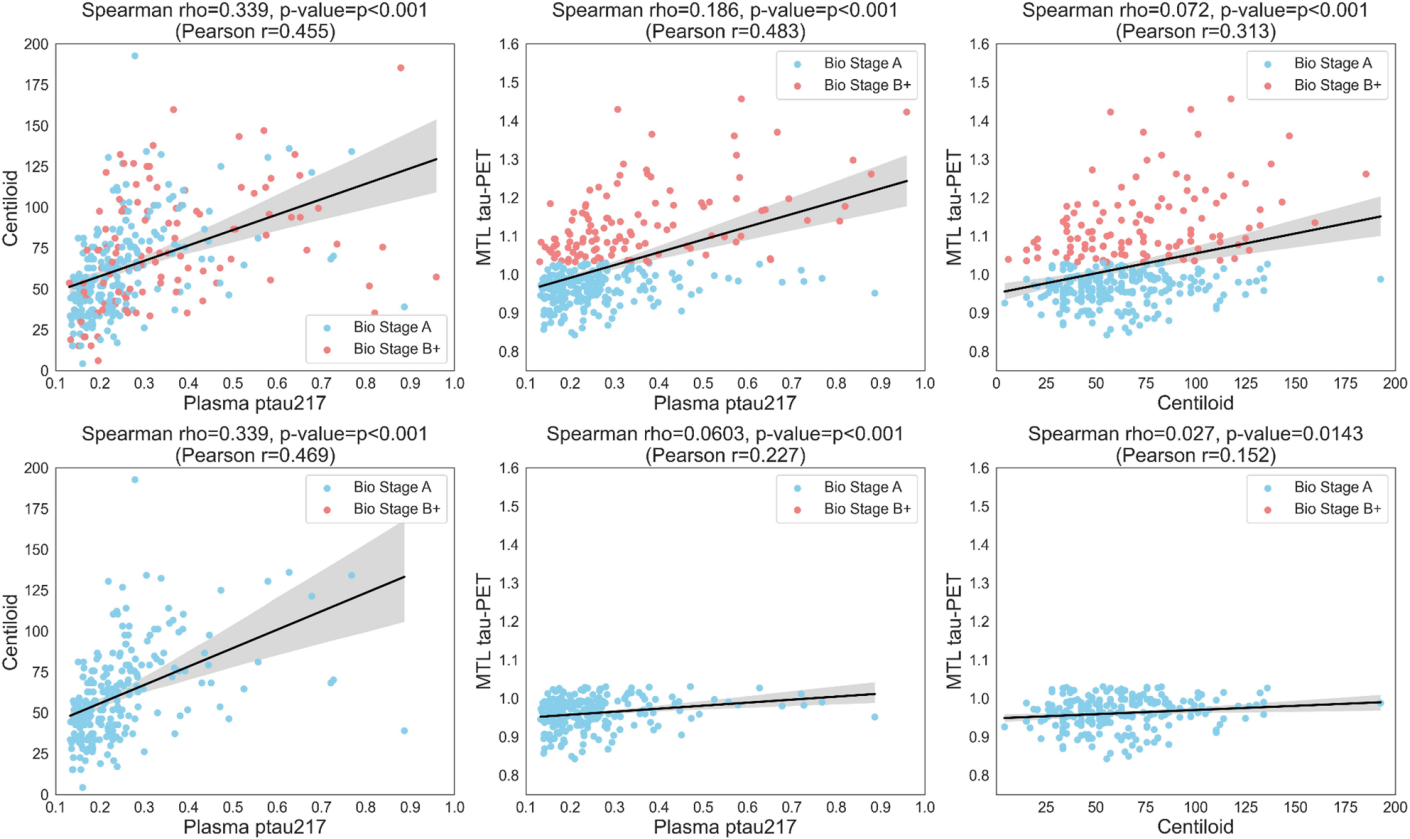
Cross sectional associations between plasma p-tau217, amyloid-PET, and MTL tau-PET. Cross sectional centiloid, entorhinal tau, and p-tau217 were all significantly correlated with each other. In the top panel, both Bio Stage A and Bio Stage B+ participants are included in the pairwise analyses. In the bottom panel, only Bio Stage A participants are included in the analyses. In our statistical analyses, Spearman rho correlations were used. For visualization purposes, we include a linear fit in the plots

Significant cross-sectional associations were present between centiloid, plasma p-tau217, and MTL tau-PET, within the full amyloid+ sample (combined Biological Stage A and B+ combined), with shared variance ranging from 0.5 to 11.6% (p-tau217 vs. centiloid: rho^2^=11.6%, rho = 0.34, *p* < 0.001; p-tau217 vs. MTL tau-PET: rho^2^=3.6%, rho = 0.19, *p* < 0.001; centiloid vs. MTL tau-PET: rho^2^=0.5%, rho = 0.07, *p* < 0.001)(Fig. 2). Cross-sectional associations remained significant when restricting to the Biological Stage A group. Compared to the full amyloid+ sample, a similar amount of variance was shared between p-tau217 and centiloid (rho^2^=11.5%, rho = 0.34, *p* < 0.001). However, the amount of variance shared between p-tau217 and MTL tau-PET (rho^2^=0.36%, rho = 0.06, *p* < 0.001) as well as between centiloid and MTL tau-PET (rho^2^=0.073%, rho = 0.027, *p* = 0.014) was reduced by more than 50% when restricted to Stage A. Cross-sectional associations of Core 1 biomarker with additional variables of interest are shown in Supplemental Results 1 (association with e4 dosage) and Supplemental Results 2 (association with inferior temporal and fusiform tau-PET SUVr).

### Prediction of tau accumulation, atrophy, and cognition in Biological Stage A

A series of LMEs were run within the Stage A group to determine whether baseline plasma p-tau217 and centiloid values were associated with subsequent tau-PET accumulation, atrophy, and cognitive performance (Supplemental Table 1).

#### Tau-PET accumulation

**Fig. 3:**
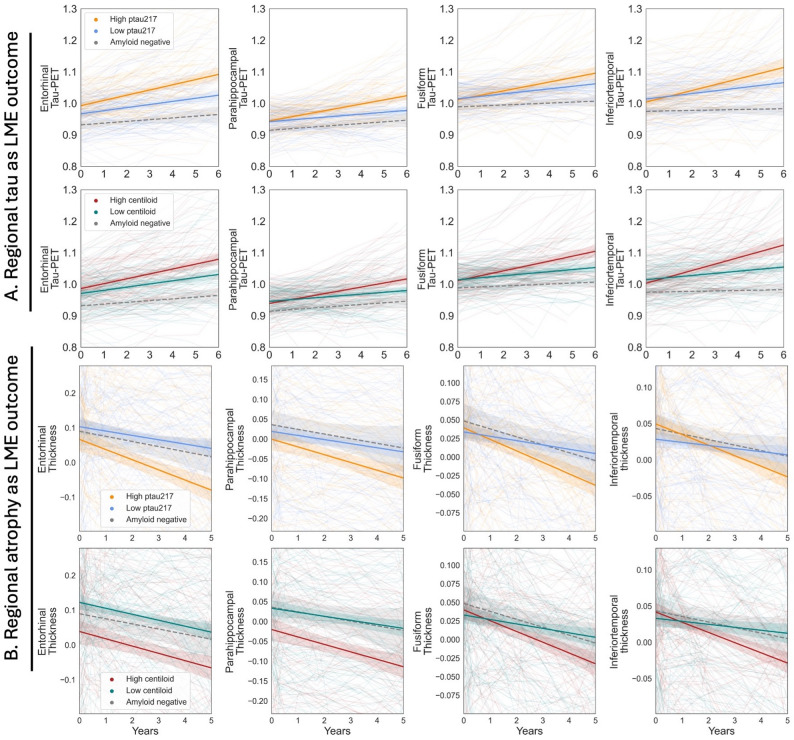
Higher p-tau217 and centiloid are associated with faster regional tau accumulation and worse atrophy. In panel (**A**), we show the relationship between plasma p-tau217 or centiloid with regional tau-PET. In panel (**B**), we show the relationship between plasma p-tau217 or centiloid with regional atrophy. In the plots, the orange and blue lines show the trajectories of high and low plasma p-tau217 participants, respectively. These groups were defined using a median split. The average p-tau217 level within the high and low p-tau217 subgroups is 0.17 and 0.33, respectively. The red and green lines show the trajectories of high and low centiloid participants, respectively. Again, a median split was used to define these groups. The average centiloid level within the high and low centiloid subgroups is 39.3 and 83.4, respectively. Binarized groups are shown here for visualization purposes, but in the LME analysis, continuous measures were used. The y-axis in panel (**B**) represents the regional thickness after regressing it onto each respective patients’ estimated total intracranial volume

Baseline plasma p-tau217 (Model 1a) was associated with higher tau accumulation in all examined regions: entorhinal (β = 0.0337, SE = 0.009, *p* < 0.001), parahippocampal (β = 0.035, SE = 0.008, *p* < 0.001), fusiform (β = 0.037, SE = 0.01, *p* < 0.001), and inferior temporal (β = 0.049, SE = 0.012, *p* < 0.001) (Fig. 3a). Likewise, baseline centiloid (Model 1b) was associated with higher tau accumulation in all regions of interest: entorhinal (β = 8.61e-5, SE = 3.84e-5, *p* = 0.026), parahippocampal (β = 1.19e-4, SE = 3.07e-5, *p* < 0.001), fusiform (β = 1.51e-4, SE = 3.95e-5, *p* < 0.001), and inferior temporal (β = 2.38e-4, SE = 4.61e-5, *p* < 0.001) (Fig. 3a). In LME models that included both baseline plasma p-tau217 and baseline centiloid as simultaneous predictors (Model 1c), plasma p-tau217 remained associated with regional tau accumulation (entorhinal, parahippocampal, and fusiform all *p* < 0.05; inferior temporal trend level *p* = 0.07). Baseline centiloid remained associated with tau accumulation in fusiform and inferior temporal (*p* < 0.05), but not significantly associated with tau accumulation in entorhinal (*p* = 0.73) and was trend level with parahippocampal (*p* = 0.07).

On visualization of Fig. 3a, it is apparent that some participants had a quadratic shape in their longitudinal tau-PET trajectories. Thus, we performed *post-hoc* tau-PET LME analyses with inclusion of an additional p-tau217*time^*2*^ or centiloid*time^*2*^ term (Supplemental Results 3). Inclusion of a p-tau217*time^*2*^ term provided limited improvement in model fits compared to the original models; in contrast, inclusion of a centiloid*time^*2*^ term provided more consistent improvements in model prediction of regional temporal tau-PET (Likelihood ratio test *p* < 0.05) and larger improvements in model Akaike Information Criterion (see Supplemental Results 3).

#### Atrophy

Baseline plasma p-tau217 (Model 1a) was associated with greater longitudinal atrophy across all regions: entorhinal (β=-0.064, SE = 0.015, *p* < 0.001), parahippocampal (β=-0.054, SE = 0.009, *p* < 0.001), fusiform (β=-0.039, SE = 0.008, *p* < 0.001), and inferior temporal (β=-0.052, SE = 0.009, *p* < 0.001) (Fig. 3b). Likewise, baseline centiloid (Model 1b) was associated with greater atrophy in all regions: entorhinal (β=-1.4e-4, SE = 6.32e-5, *p* = 0.028), parahippocampal (β=-1.25e-4, SE = 3.86e-5, *p* = 0.001), fusiform (β=-1.26, SE = 3.09e-5, *p* = 6.42e-5), and inferior temporal (β=-1.78e-4, SE = 3.55e-5, *p* = 1.31e-6) (Fig. 3b). In models that included both baseline plasma p-tau217 and centiloid as predictors (Model 1c), plasma p-tau217 remained significantly associated with greater atrophy in all four regions (*p* < 0.05). Baseline centiloid remained associated with inferior temporal atrophy (*p* = 0.01), but was no longer associated with entorhinal (*p* = 0.83) or parahippocampal (*p* = 0.62) atrophy. The association between centiloid and fusiform was trend level (*p* = 0.07).

#### Cognitive performance

**Fig. 4:**
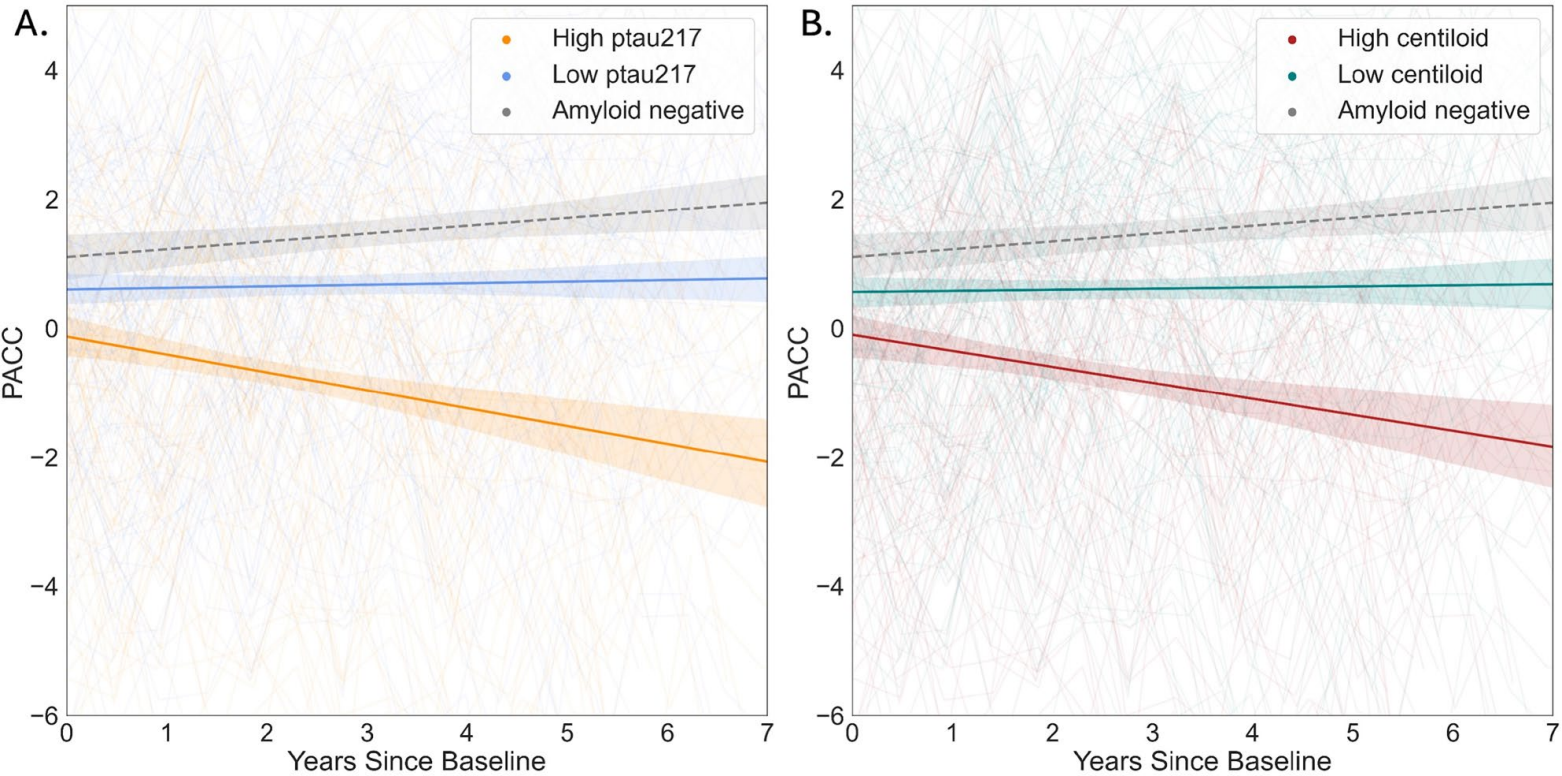
Higher p-tau217 and centiloid are associated with worse cognitive trajectory. In panel (**A**), we show the relationship between baseline plasma p-tau217 with longitudinal PACC. In panel (**B**), we show the relationship between baseline centiloid with longitudinal PACC. As was the case in Fig. 3, the orange and blue lines show the trajectories of high and low plasma p-tau217 participants, respectively. The red and green lines show the trajectories of high and low centiloid participants, respectively. Again, these were defined by median splits. Binarized groups are shown here for visualization purposes, but in the LME analysis, continuous measures were used

In separate models, baseline plasma p-tau217 (Model 1a; β=-1.65, SE = 0.43, *p* < 0.001) and baseline centiloid (Model 1b; β=-0.006, SE = 0.002, *p* < 0.001) were associated with worse longitudinal PACC performance (Fig. 4). When modelled together (Model 1c) both baseline plasma p-tau217 (β=-1.16, SE = 0.49, *p* = 0.019) and centiloid (β=-0.004, SE = 0.002, *p* = 0.031) values remained significantly associated with worse longitudinal PACC scores.

#### Sensitivity analyses

We ran additional sensitivity LME analyses to explore models with different covariates (e.g., inclusion of treatment arm term). We also explored alternative approaches to defining the Biological Stage A (e.g., lowering the MTL tau-PET positivity threshold further; as well as further exclusion of participants that were below the MTL threshold, but had focal elevations in other cortical regions) (Supplemental Results 4–6, Supplemental Table 2). In summary, the following patterns emerged across these sensitivity analyses. As was the case for the primary analysis, baseline plasma p-tau217 was consistently associated with longitudinal outcomes, both when modeled alone in LME analyses and when modeled with centiloid. Further, centiloid was also associated with longitudinal outcomes, but the effect was overall weaker compared to p-tau217, in particular for entorhinal and parahippocampal tau-PET and atrophy, where it tended to be non-significant, mirroring our primary analysis (see Supplemental Table 2 for full model outputs). Finally, the effect of baseline p-tau217 and centiloid on longitudinal PACC was trend level or non-significant in a subset of sensitivity analyses.

### Plasma p-tau217 and centiloid are associated with higher risk of progression to Biological Stage B+

**Fig. 5:**
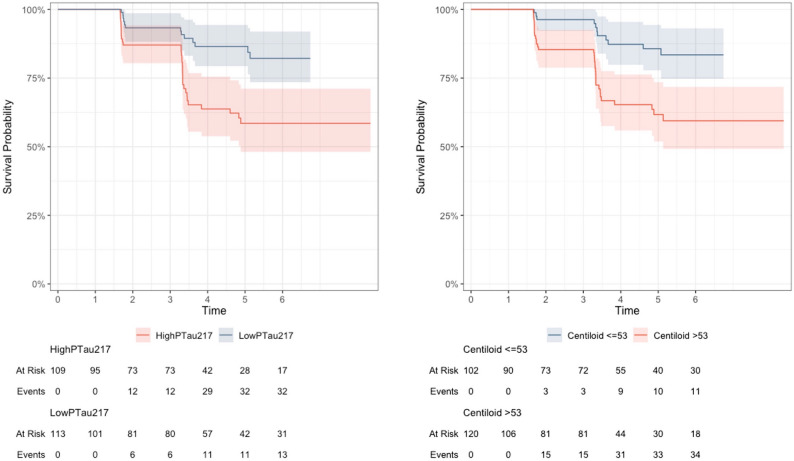
Higher p-tau217 and centiloid are associated with higher risk of conversion to Biological Stage B. Graphs show the Kaplan-Meier estimates for survival based on p-tau217 (left panel) or centiloid (right panel). High and low plasma p-tau217 groups, and high and low centiloid groups were defined using median splits. In the Cox proportional hazards analyses, continuous, rather than binary, measures of p-tau217 and centiloid were used

A total of 48 Biological Stage A participants (22%) progressed to Biological Stage B+ during the course of follow-up (mean follow up = 4.2 years; Fig. 5). When examined in separate models, p-tau217 (HR = 1.81, CI=(1.41–2.32), *p* < 0.001) and centiloid (HR = 1.24, CI=(1.12–1.38), *p* < 0.001) were associated with increased progression to Biological Stage B+. Both p-tau217 and centiloid values were independently associated with progression to Biological Stage B+ when included in the same model (p-tau217: HR = 1.49, CI=[1.10–2.03], *p* = 0.01; centiloid: HR = 1.15, CI=[1.03–1.30], *p* = 0.02).

## Discussion

In this study, we implemented the recently proposed 2024 criteria for the diagnosis and staging of AD to identify and characterize a group of CU individuals in Biological Stage A. This is the earliest proposed biological stage of AD detectable with current biomarkers, reflecting a point in which early pathological processes have been initiated. More specifically, this stage can be defined by positivity in “Core 1” early changing markers, such as amyloid-PET or plasma p-tau217, in conjunction with negativity in downstream tau markers that are indicative of disease progression. Within this early Stage A group, we sought to establish whether variability in measures of early changing Core 1 markers were biologically relevant. We found that both baseline amyloid-PET and plasma p-tau217 were associated with longitudinal accumulation in tau-PET, greater atrophy, and worse cognitive performance. Complementing these longitudinal models, we also found that elevated baseline amyloid-PET and plasma p-tau217 were also associated with faster progression to Biological Stage B+ (MTL tau-PET positivity). Our findings highlight that variability within Core 1 AD biomarkers provides important prognostic information during the earliest stage of biologically defined AD.

Our finding that levels of amyloid-PET and plasma p-tau217 are biologically meaningful is consistent with other studies. Within CU individuals, multiple studies have shown that amyloid-PET signal (even within the A+ range) is associated with longitudinal cognitive performance or clinical progression to MCI or dementia [9, 29, 30]. Similarly, studies have also demonstrated that within CU A+ study participants, elevated levels of baseline plasma p-tau217 predicts longitudinal cognitive performance and clinical progression [11, 13, 14]. However, these aforementioned studies did not restrict analyses to the subgroup of amyloid + CU individuals that were also negative on tau-PET, to establish whether the continuum of amyloid-PET and/or plasma p-tau217 remains biologically relevant. This is especially important to distinguish, as tau-PET + CU are known to be at increased risk of future tau accumulation [31, 32] and clinical progression [33, 34]. Our approach addresses this question, and additionally places findings in the context of the revised biological criteria for the diagnosis and staging of AD [2]. Our results demonstrate that even among the earliest biologically defined stage of AD (Stage A), the continuum of both PET and plasma measures of early AD processes are predictive of multiple disease relevant changes over time. A recent study likewise evaluated longitudinal tau-PET trajectories among participants who were deemed tau-PET negative at baseline in two cohorts of aging, Mayo Clinic Study of Aging and BioFinder 2 [35]. In line with our findings, they also demonstrated predictive capabilities of Core 1 biomarkers on longitudinal tau-PET. Our study further extends these findings by showing prognostic capabilities of these markers on downstream atrophy and neuropsychological performance, all in an independent cohort.

Plasma p-tau217 is among the newest of the staging biomarkers proposed for use in the 2024 criteria, and one assay measuring p-tau217 (different from the assay used in A4) has recently gained FDA clearance (as a ratio with plasma amyloid-β 42) [36]. Early studies on plasma p-tau217 showed significant associations with established markers of amyloid and tau (i.e., CSF and PET) [37–40]. Further, multiple studies have demonstrated that plasma p-tau217 may be more strongly associated with amyloid than tau particularly early in the disease course [37, 38], and therefore may become abnormal earlier in the disease course [41]. Given these early-changing characteristics, p-tau217 has been proposed to be a Core 1 biomarker in the revised criteria, to determine whether someone is on the AD spectrum (Stage A+) but not as a prognostic measure (Stages B-D). Plasma p-tau217 and amyloid-PET measures are strongly correlated, with AUCs typically exceeding 0.90 in discrimination analyses using p-tau217 to predict amyloid-PET positivity [40]. As a practical application, in the context of clinical trial screening, p-tau217 has been demonstrated to have promise for enriching preclinical AD trials for participants who are amyloid positive by PET imaging [27, 42–44]. Overall, close coupling between p-tau217 and amyloid-PET was recapitulated in our analyses even after restricting to the Stage A group. However, the shared variance between these two Core 1 measures was only around 11.6% (if using Pearson r-squared, rather than Spearman rho-squared, shared variance still only remains 23%) among our amyloid + CU participants, and these measures likely reflect distinct aspects of early disease biology. Our finding that both centiloid and p-tau217 values were independently associated with multiple downstream markers highlights that each measure contains meaningful prognostic information within the earliest stage of AD.

While the associations of plasma p-tau217 and centiloids with downstream markers was relatively consistent across our analyses, patterns emerged illustrating varying effects across predictors and outcomes. First, in our LME analyses that included both centiloid and p-tau217 as simultaneous predictors, centiloids were not associated with longitudinal tau-PET or atrophy in the medial temporal regions whereas p-tau217 was a significant independent predictor. Recent work has shown that the general ordering of detectable biomarker abnormalities is amyloid-PET, then plasma ptau217, then MTL tau-PET, then neocortical tau [37, 41, 45, 46]. In this context, it is possible that the effects of baseline centiloid on downstream AD markers is mediated by plasma p-tau217. Data from other studies examining a broader range of participants beyond just Biological Stage A supports such a model [37, 39, 41, 47]. An additional interpretation is that the effect of amyloid-PET on downstream changes at this biological stage may be non-linear. Our post-hoc LME tau-PET analyses, where we included a quadratic term in the models, lend credence to this view. In these analyses (Supplemental Results 3), addition of an interaction term between amyloid-PET and quadratic time led to consistent significant improvements in model prediction of longitudinal regional tau-PET. This was the case for both when centiloid was the sole biomarker predictor in the model and when the model included plasma p-tau217. Notably, the improvement with quadratic modeling was seen in prediction of longitudinal parahippocampal tau-PET, where the effect of amyloid-PET was originally diminished when modeled together with plasma p-tau217 in the linear-time approach. These analyses are overall supportive of the likely non-linear effect of baseline centiloid level on the rate of change of tau-PET in temporal regions, an interpretation which has also been reported recently in alternative cohorts [48].

Another pattern that emerged was that the effects of baseline Core 1 biomarker level on longitudinal tau PET accumulation and neurodegeneration was overall stronger than their effects on longitudinal cognition (see Supplemental Tables 1 and Supplemental Table 2 for primary and sensitivity analyses model outputs, respectively). This pattern of findings may reflect the time delay between Core 1 biomarker and neuropsychological changes being longer than the time delay between Core 1 biomarker and tau-PET/atrophy changes. Longer term follow-up may be better powered to discern the effects of Core 1 biomarkers on longitudinal cognitive decline. Further, that the effect of Core 1 markers on downstream changes may vary by different subsets on participants (i.e., variability across sensitivity analyses in which different thresholds were applied to define Biological Stage A) highlights an important practical point. To date, there have been major multi-site efforts to standardize the definition of amyloid positivity (e.g., centiloid) for inclusion into the AD continuum. However, work is ongoing to define tau-PET positivity and staging thresholds [8, 49–52]. We iterated across multiple Biological Stage A definitions and overall demonstrated relative stability in findings, especially when predicting downstream tau accumulation and atrophy; nevertheless, further work on standardization and validation of tau-PET staging thresholds for operationalization of the updated staging criteria is needed, especially during the earliest stages of AD (also discussed in the limitations below).

In the revised criteria for the diagnosis and staging of AD, the workgroup explicitly states that the framework is meant to be applied only to those with cognitive impairment; further, they recommend that the staging be used for CU only in the context of research studies including clinical trials or observational studies. With a large focus in the field on secondary prevention (e.g., AHEAD 3–45 [53], TRAILBLAZER 3 [NCT05026866], and SKYLINE [NCT05256134]), it is of critical importance to understand how this framework applies in the earliest biological stages in amyloid PET positive individuals without cognitive impairment. These findings are particularly relevant for trials aiming to move even earlier in the pathophysiological sequence, such as the AHEAD A3 trial at the intermediate levels of amyloid PET, and future trials getting closer to primary prevention of amyloid “positivity”. If a prevention trial is successful, medical practice will need to rapidly adapt and integrate biological staging into medical decision making for CU individuals. In addition to Core 1 biomarkers potentially being used in the future to confirm that an individual is on the AD neuropathological spectrum, our findings suggest that additional information leveraging the continuum of amyloid-PET and plasma p-tau217 values may be clinically relevant beyond just classification into Stage A. Future work in a real-world setting is critical to establish whether Core 1 biomarkers may be leveraged in a manner that provides individuals classified as Stage A with additional prognostic information.

Our study has multiple limitations. First, while it is among the first to apply the new criteria to a large sample of CU older adults, the sample here is not representative of the general population given the high education in the cohort and the setting of a secondary prevention trial. Next, we defined Biological Stage A+ based on amyloid-PET, whereas plasma p-tau217 can also be used for making this classification according to the new criteria. We focused on amyloid-PET positivity to define Stage A given that this classification was used for enrollment into A4 and involved a hybrid quantitative-qualitative method. If attempting to define Biological Stage A+ based on plasma p-tau217, rather than amyloid-PET, it would be ideal to have representation of amyloid-PET negative participants with plasma p-tau217 data. Given the focus of amyloid PET+ individuals into A4, this current dataset was not appropriate to address alternative definitions using plasma ptau217. More to the point of defining the Biological Stages, as discussed above, there is not yet a firmly established approach for defining MTL tau-PET positivity (i.e., the threshold between Biological Stage A and B). In our study, we used GMM, an approach that has been widely used for defining amyloid-PET and tau-PET thresholds [8, 39, 50, 51, 54–57], and demonstrated relative stability across thresholds in our findings through sensitivity analyses. Notably, in Fig. 2, there remains a subtle association between plasma p-tau217 and MTL tau-PET, as well as between centiloid and MTL tau-PET. This observation does suggest that subtle elevations in MTL tau PET may indicate early tau burden, consistent with prior studies showing biological relevance of tau PET SUVrs that would fall below traditional threshold approaches (as an example, see Supplemental Table 3 in [58]). Thus, although these cases fall below the conservative threshold used in our study, biologically meaningful signal may still exist in this range (and likely reflects a mixture of true elevated signal and noise that results in slightly elevated values). Limitations with threshold selection is not specific to our study and in fact reflects a general limitation in operationalizing the biological staging criteria. Beyond threshold selection, biological staging may also be affected by PET tracer selection. While we were able to show stability across thresholds in our sensitivity analyses, we are unable to examine in A4 whether the observed effects of Core 1 markers on subsequent change in regional tau-PET may differ with second generation tracers, such as MK-6240.

## Conclusion

Among CU participants in the earliest biological stage of AD, levels of amyloid-PET and plasma p-tau217 predicted longitudinal change in downstream AD markers. This work highlights the potential for future strategies that leverage multiple early changing biomarkers of AD to inform individual level risk of future progression among CU.

## Supplementary Information

Supplementary Material 1: Supplemental Table 1.

Supplementary Material 2: Supplemental Table 2.

Supplementary Material 3: Supplemental Materials.

## Data Availability

All data produced in the present work are derived from public datasets that are available at https://www.a4studydata.org/and https://www.synapse.org/a4_learn_datasharing/ after user registration.
