# Supplementary material for: Continuum of Core 1 biomarkers in preclinical Alzheimer’s disease
Source: Alzheimers Res Ther. 2026 Apr 10;18:133. doi: 10.1186/s13195-026-02044-1 (PMC13220391; doi:10.1186/s13195-026-02044-1)
Supplement: Supplementary file 3 — Supplementary Material 3: Supplemental Materials. [file 13195_2026_2044_MOESM3_ESM.docx]

**Continuum of Core 1 Biomarkers in Preclinical Alzheimer’s Disease**

Supplemental Methods 1. Calculation of tau-PET MTL SUVr.

Supplemental Methods 2. Derivation of time variable for linear mixed effects models analyses.

Supplemental Results 1. Association between APOE e4 dosage and Core 1 biomarkers.

Supplemental Results 2. Association between Core 1 biomarkers and inferior temporal or fusiform tau-PET.

Supplemental Results 3. Longitudinal tau-PET analyses with quadratic time term in linear mixed effects models.

Supplemental Results 4. Sensitivity analyses using alternative biomarker thresholds and model covariates.

Supplemental Results 5. Average SUVr by Braak region.

Supplemental Results 6. Regional tau-PET SUVr distributions.

Supplemental Table 1. Model estimates from primary longitudinal tau, MRI, and PACC LME analyses

Supplemental Table 2. Model estimates from sensitivity analyses with differing definitions of Biological Stage A and with different covariates

References

**Supplemental Methods 1. Calculation of tau-PET MTL SUVr.**

To split the A4 sample into Biological Stage A and Biological Stage B+ (Stage B and beyond), we applied a Gaussian mixture model approach on the distribution of tau-PET MTL SUVrs. The tau-PET MTL SUVrs were defined as follows. For each participant, we extracted the regional SUVrs from bilateral entorhinal and parahippocampal cortex (variables *entorhinal_lh, entorhinal_rh, parahippocampal_lh, parahippocampal_rh*, spreadsheet *imaging_SUVR_tau*.csv) from their first tau-PET scan. We then calculated the MTL SUVr as the weighted average from these regional SUVrs. The weights are regional volumes derived from GTMseg, a FreeSurfer-based MNI-space atlas that was produced from averaging regional segmentations from the participants of the Harvard Aging Brain study. This was the same atlas from which the regional SUVrs above were derived. The atlas is available for download from <https://habs.mgh.harvard.edu/researchers/data-tools/>. Because this template atlas was used, the weights reflect atlas regional volumes, rather than A4 participant-level volumes.

**Supplemental Methods 2. Derivation of time variable for linear mixed effects models analyses.**

For tau-PET, MRI, and PACC data available on the A4 data portal, timing information is coded as a visit code *VISCODE* variable. This *VISCODE* variable had to be transformed into a time measurement for linear mixed effects models analyses. We linked the *VISCODE* variable with the days since consent *SVSDTC_DAYS_CONSENT* variable in the *SV.csv* spreadsheet. This linkage allowed us to calculate days between scans or PACC administrations. For tau-PET and MRI analyses, the *time* variable in the linear mixed effects models represents time since their first tau-PET or MRI scan. For PACC analyses, the *time* variable in the linear mixed effects models represents time since their baseline PACC administration.

There were 37 tau-PET scans that occurred at an “early termination” visit (*VISCODE* value 999) and had no corresponding days since consent *SVSDTC_DAYS_CONSENT* value. For these scans, we used the days since consent *SVSDTC_DAYS_CONSENT* value from *VISCODE* 997 (which corresponds to open label extension [early termination or study close]) as an estimate of time.

There were 5 tau-PET scans that occurred at other visits (*VISCODE* 27, 48, and 84) but had no days since consent *SVSDTC_DAYS_CONSENT.* To derive a time value for these scans, we took the *SVSDTC_DAYS_CONSENT* value from the prior *VISCODE* that had a corresponding *SVSDTC_DAYS_CONSENT* value. We then added the expected number of days based on the study schedule to get to the *VISCODE* of interest (see Table below for example calculation and for summary of these 5 cases; adapted from Young et al., 2026, *in preparation*).

| **BID** | **Missing *VISCODE*** | **Weeks at *VISCODE* (based on study schedule)** | **Prior *VISCODE* with corresponding *SVSTDTC_ DAYS_ CONSENT*** | **Weeks at prior *VISCODE* (based on study schedule)** | ***SVSTDTC_ DAYS_ CONSENT* at prior *VISCODE*** | **Estimated *SVSTDTC_ DAYS_ CONSENT*** |
| --- | --- | --- | --- | --- | --- | --- |
| B18613471 | 84 | 312 | 80 | 296 | 2353 | 2353 + (312 - 296)*7 = 2465 |
| B32880198 | 84 | 312 | 83 | 308 | 2358 | 3086 |
| B69161824 | 48 | 168 | 47 | 164 | 1310 | 1338 |
| B86248441 | 84 | 312 | 82 | 304 | 2239 | 2295 |
| B90395301 | 27 | 84 | 26 | 80 | 632 | 1248 |


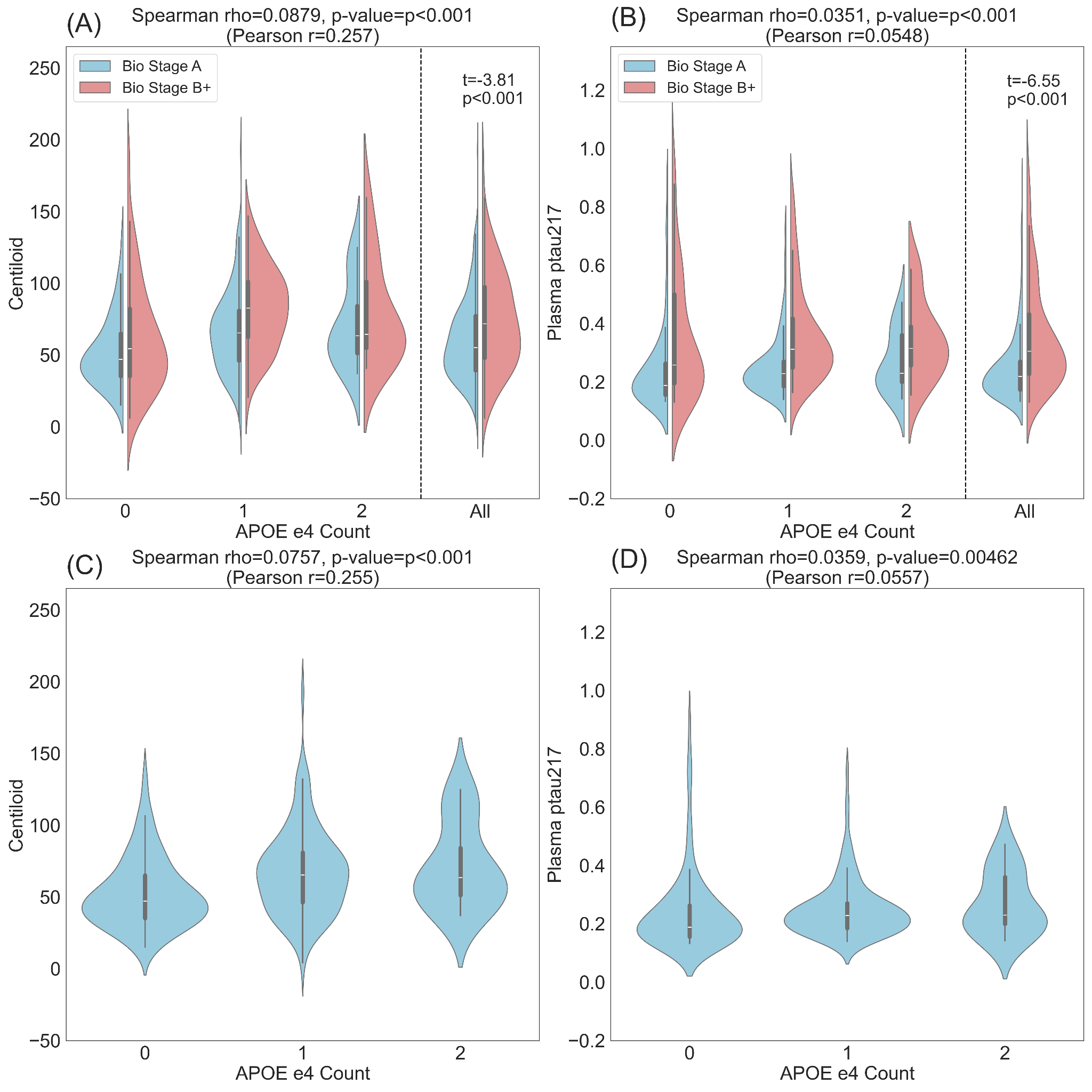


**Supplemental Results 1. Association between APOE e4 dosage and Core 1 biomarkers.** In (A) and (B), we show the Centiloid and plasma p-tau217 distributions for e4 non-carriers, heterozygotes, and homozygotes, separately for Biological Stage A and B+. The Spearman rho and Pearson r describe the association between e4 dosage and Centiloid or plasma p-tau217 for the combined (Biological Stage A and B+) group. For reference, in (A) and (B), we also show Centiloid and plasma p-tau217 distributions for Biological Stage A and B+, aggregated across all the genotypes (labeled All). The t-statistic and p-value in the upper right of (A) and (B) represent the t-test difference in Centiloid or plasma p-tau217 between Biological Stage A or B+. In (C) and (D), we show the Centiloid and plasma p-tau217 distributions for e4 non-carriers, heterozygotes, and homozygotes only for the Biological Stage A participants. The Spearman rho and Pearson r above these plots describe the association between e4 dosage and Centiloid or plasma p-tau217 within the Biological Stage A group alone. While increasing e4 dosage is associated with increasing Centiloid or plasma p-tau217 level, the shared variance between them is modest.


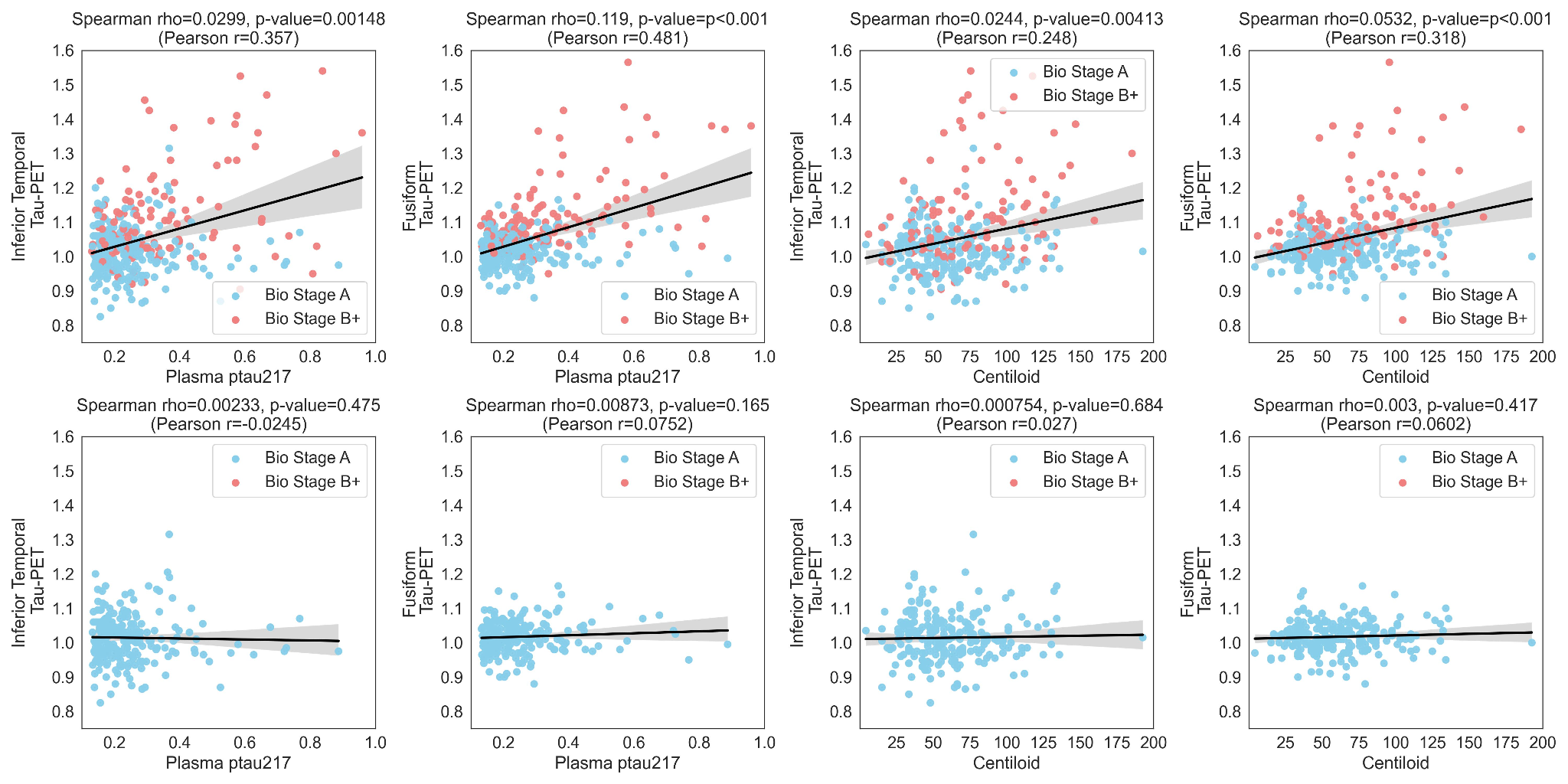


**Supplemental Results 2. Association between Core 1 biomarkers and inferior temporal or fusiform tau-PET SUVr.** In the top panel, both Bio Stage A and Bio Stage B+ participants were included in the pairwise correlation analyses that are shown above the plots. In the bottom panel, only Bio Stage A participants were included in the pairwise correlation analyses that are shown above the plots. Core 1 biomarker level is modestly associated with inferior temporal and fusiform tau-PET SUVr when examining the entire amyloid+ group; this association was not present when restricting the analyses to the Bio Stage A participants.

**Supplemental Results 3. Longitudinal tau-PET analyses with quadratic time term in linear mixed effects models.**

Visualization inspection of Figure 3A (longitudinal tau-PET analyses) demonstrates that some participants may have a quadratic shaped trajectory of their regional tau-PET SUVrs. Because of this, we re-performed the longitudinal tau-PET analysis with inclusion of a quadratic time term (e.g., (*p-tau217*time^2^* or *centiloid*time^2^*) in the Model 1a, Model 1b, and Model 1c analyses (all of the lower level terms remained included). We refer to these updated models (that have the additional *biomarker*time^2^* term) as Model 1a quad, Model 1b quad, and Model 1c quad in the table below. The table shows the estimates and p-values for the *biomarker*time* and *biomarker*time^2^* terms from each of the updated models.

In each instance, we used a likelihood ratio test (LRT) to test whether the updated model with the quadratic term (e.g., Model 1a quad) is significantly different from the original model without the quadratic term (e.g., original Model 1a from main manuscript). We then also report the change in model Akaike Information Criterion after addition of the quadratic terms (AIC delta), with a lower AIC delta indicating better model fit. The model with the quadratic time term was considered a better fit than the model with only the linear time term if the p-value from the LRT was less than 0.05.

Across models including p-tau217 alone (Model 1a quad.), inclusion of a quadratic time interaction term provided limited improvement in model fit for tau PET regions, with evidence for nonlinearity observed only in the parahippocampal and fusiform cortex. By contrast, models including amyloid burden alone (Model 1b quad.) demonstrated significant *centiloid*time^2^* interactions in parahippocampal, fusiform, and inferior temporal regions, accompanied by substantial improvements in model fit.

In models simultaneously including both p-tau217 and amyloid (Model 1c quad), quadratic effects associated with amyloid (*centiloid*time^2^*) remained significant in multiple tau PET regions, whereas *p-tau217*time^2^* terms were not significant. Across these models, the inclusion of quadratic time terms significantly improved model fit compared to the linear only biomarker time models. These findings indicate that allowing nonlinear biomarker–time relationships improves characterization of longitudinal tau-PET change, particularly for amyloid-related effects in temporal regions.

***(Model 1a quad.) Regional tau-PET ~ p-tau217*time + p-tau217*time^2^ + covariates*time + random intercept and slope***

| **Regional tau-PET outcome** | ***p-tau217*time*** | ***p-tau217*time^2^*** | **LRT p-value** | **AIC delta** |
| --- | --- | --- | --- | --- |
| Entorhinal | 4.49e-2 (1.71e-2); p=0.009 | -1.50e-3 (2.62e-3); p=0.57 | 0.57 | 1.75 |
| Parahippocampal | 7.11e-3 (1.36e-2); p=0.60 | 5.04e-3 (2.08e-3); p=0.02 | **0.02** | -3.78 |
| Fusiform | 2.77e-3 (1.47e-2); p=0.86 | 6.51e-3 (2.07e-3); p=0.002 | **0.002** | -7.77 |
| Inferior temporal | 2.78e-2 (1.78e-2); p=0.12 | 3.95e-3 (2.56e-3); p=0.12 | 0.12 | -0.29 |

LRT p-value: Represents the p-value in a test comparing the updated model (with the *p-tau217*time^2^* term) to the original linear time model from the main manuscript (for reference, all estimates and p-values from our original linear mixed effects models can be found in the separate Supplemental Table 1 file). AIC delta: Represents the change in AIC after addition of the *p-tau217*time^2^*; lower value indicates improved fit.

***(Model 1b quad.) Regional tau-PET ~ centiloid*time + centiloid*time^2^ + covariates*time + random intercept and slope***

| **Regional tau-PET outcome** | ***centiloid*time*** | ***centiloid*time^2^*** | **LRT p-value** | **AIC delta** |
| --- | --- | --- | --- | --- |
| Entorhinal | 4.02e-5 (6.56e-5); p=0.54 | 8.57e-6 (9.77e-6); p=0.38 | 0.38 | 1.31 |
| Parahippocampal | -2.80e-5 (5.17e-5); p=0.59 | 2.71e-5 (7.70e-6); p=0.0004 | **0.0005** | -10.16 |
| Fusiform | -3.26e-5 (5.58e-5); p=0.56 | 3.48e-5 (7.62e-6); p=6.43e-6 | **6.39e-6** | -18.28 |
| Inferior temporal | 8.87e-5 (6.74e-5); p=0.19 | 2.85e-5 (9.48e-6); p=0.003 | **0.003** | -6.81 |

LRT p-value: Represents the p-value in a test comparing the updated model (with the *centiloid*time^2^* term) to the original linear time model from the main manuscript. AIC delta: Represents the change in AIC after addition of the *centiloid*time^2^*; lower value indicates improved fit.

***(Model 1c quad.) Regional tau-PET ~ p-tau217*time + centiloid*time + p-tau217*time^2^ +***

***centiloid*time^2^ + covariates*time + random intercept and slope***

| **Regional tau-PET outcome** | ***p-tau217*time*** | ***centiloid*time*** | ***p-tau217*time^2^*** | ***centiloid*time^2^*** | **LRT p-value** | **AIC delta** |
| --- | --- | --- | --- | --- | --- | --- |
| Entorhinal | 5.41e-2 (2.00e-2); p=0.007 | -6.41e-5 (7.55e-5); p =0.40 | -3.74e-3 (3.15e-3); p=0.23 | 1.55e-5 (1.17e-5); p=0.19 | 0.36 | 2.15 |
| Parahippocampal | 1.87e-2 (1.57e-2); p=0.23 | -6.50e-5 (5.96e-5); p=0.28 | 1.14e-3 (2.50e-3); p=0.65 | 2.43e-5 (9.23e-6); p=0.009 | **0.002** | -7.88 |
| Fusiform | 1.43e-2 (1.68e-2); p=0.39 | -5.99e-5 (6.44e-5); p=0.35) | 1.60e-3 (2.47e-3); p=0.52 | 3.10e-5 (9.21e-6); p=0.0008 | **3.89e-5** | -16.11 |
| Inferior temporal | 2.80e-2 (2.03e-2); p=0.17 | 3.46e-5 (7.80e-5); p=0.66 | -1.02e-3 (3.08e-3); p=0.74 | 3.01e-5 (1.15e-5); p=0.009 | **0.013** | -4.51 |

LRT p-value: Represents the p-value in a test comparing the updated model (with the *p-tau217*time^2^* and *centiloid*time^2^* term) to the original linear time model from the main manuscript. AIC delta: Represents the change in AIC after addition of the *p-tau217*time^2^* and *centiloid*time^2^* terms; lower value indicates improved fit.

**
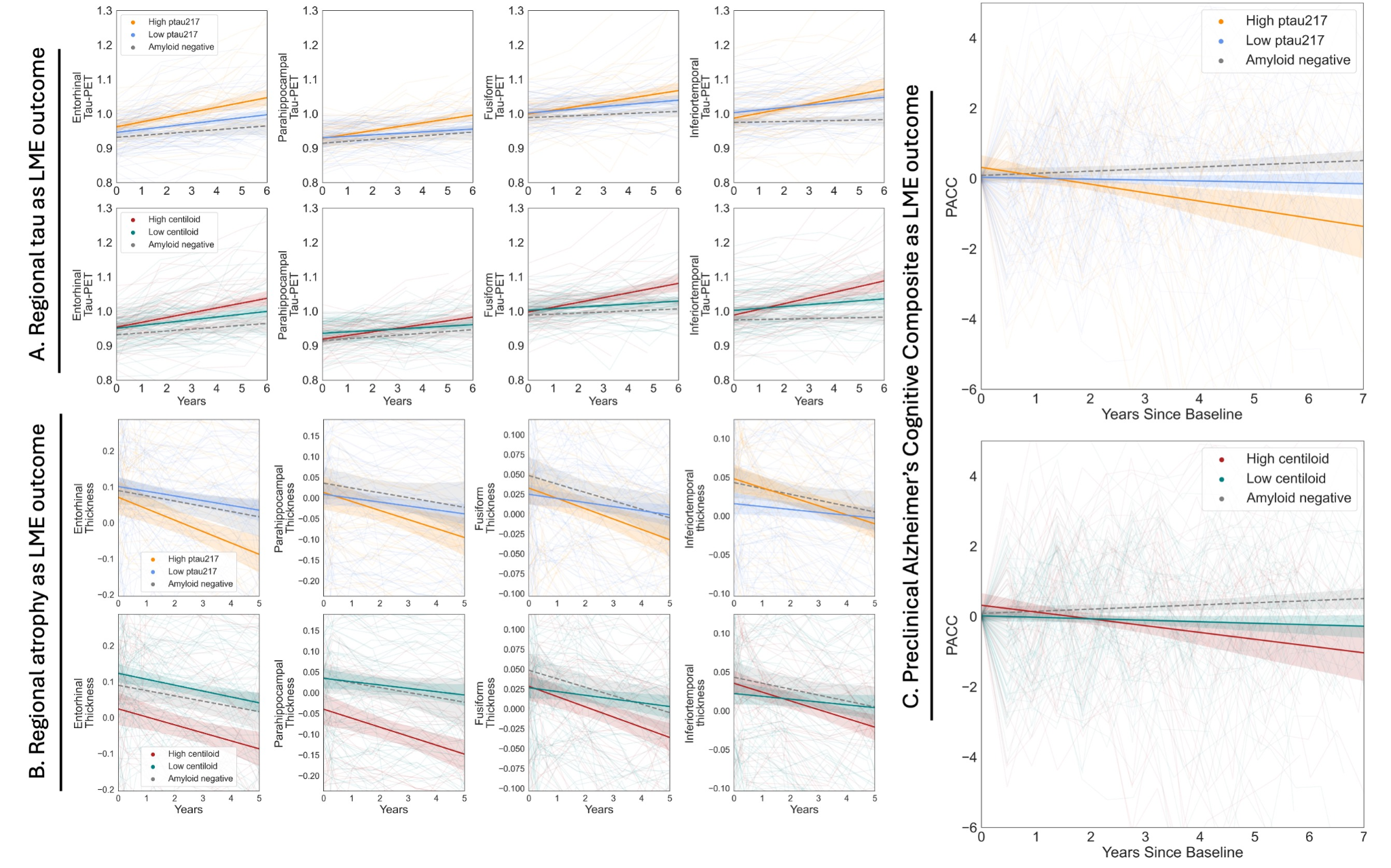
**

**Supplemental Results 4. Sensitivity analyses using alternative biomarker thresholds and model covariates.** In this sensitivity analysis, we used a threshold of 1 SD (rather than 1.5 SD) above the mean of the first component (tau-PET negative) identified by GMM to split the A4 sample into Biological Stage A and Biological Stage B+. We tested this more conservative threshold to ensure MTL tau-PET+ cases were excluded from Biological Stage A (i.e., to ensure our results were not being driven by a few subjects that had relatively higher MTL tau-PET+. In panel (A), we show the relationship between plasma p-tau217 or centiloid with regional tau-PET. In panel (B), we show the relationship between plasma p-tau217 or centiloid with regional atrophy. In panel (C) we show the relationship between plasma p-tau217 or centiloid. In the plots, the orange and blue lines show the trajectories of high and low plasma p-tau217 participants, respectively. These groups were defined using a median split. The red and green lines show the trajectories of high and low centiloid participants, respectively. Again, a median split was used to define these groups. Binarized groups are shown here for visualization purposes, but in the LME analysis, continuous measures were used. Model estimates are shown in Supplemental Table 2.

Supplemental Table 2 also has model estimates from additional sensitivity analyses as follows. There are several participants that were included in A4 (deemed to have a positive amyloid-PET scan visually), but ultimately had a low calculated Centiloid level; we ran sensitivity LME analyses, where we excluded from our LME analyses participants that had a baseline Centiloid<=20.0 (8 participants excluded; 214 participants left for analysis). Further, in our primary analyses, we excluded 23 participants with p-tau217 data below the limits of the assay, but with available raw data; we ran a sensitivity LME analyses, where we included the 23 participants with p-tau217 levels below the lower limit of the assay. Additionally, we re-performed the LME analyses with a term to indicate treatment arm (Solanezumab or Placebo). Lastly, it is known that there are participants with temporal-sparing tau-PET patterns in the A4 study [(1)](https://paperpile.com/c/TJ3uCs/U8oAV). Because the delineation between Biological Stage A and B is based on MTL tau-PET (as is suggested in the updated criteria [(2)](https://paperpile.com/c/TJ3uCs/AQUXC)), we examined whether any of our Biological Stage A participants had elevated tau-PET signal in mid- to late-Braak ROIs (Supplemental Results 5). As noted below, although there are Biological Stage B+ participants with elevated average SUVr in mid- to late- Braak ROIS (Braak 3-6), there is no indication that this is the case for the Biological Stage A group. To evaluate this further, we selected 23 Biological Stage A participants that had elevated tau-PET SUVr in at least one ROI (see Supplemental Results 6). We excluded these 23 participants and re-ran sensitivity LME analyses, whose estimates are shown in Supplemental Table 2.

To summarize the overall findings from our sensitivity analyses, the primary patterns that emerged were: (1) baseline ptau217 was consistently associated with longitudinal outcomes; (2) centiloid was more weakly associated with longitudinal outcomes, such that when modeled together with p-tau 217, its effect on tau-PET/atrophy in medical temporal regions, and PACC varies (trend-level / non-significant for subsets of sensitivity analyses); (3) the effects on Core 1 biomarkers on longitudinal PACC was trend level or non-significant in a subset of sensitivity analyses.

**
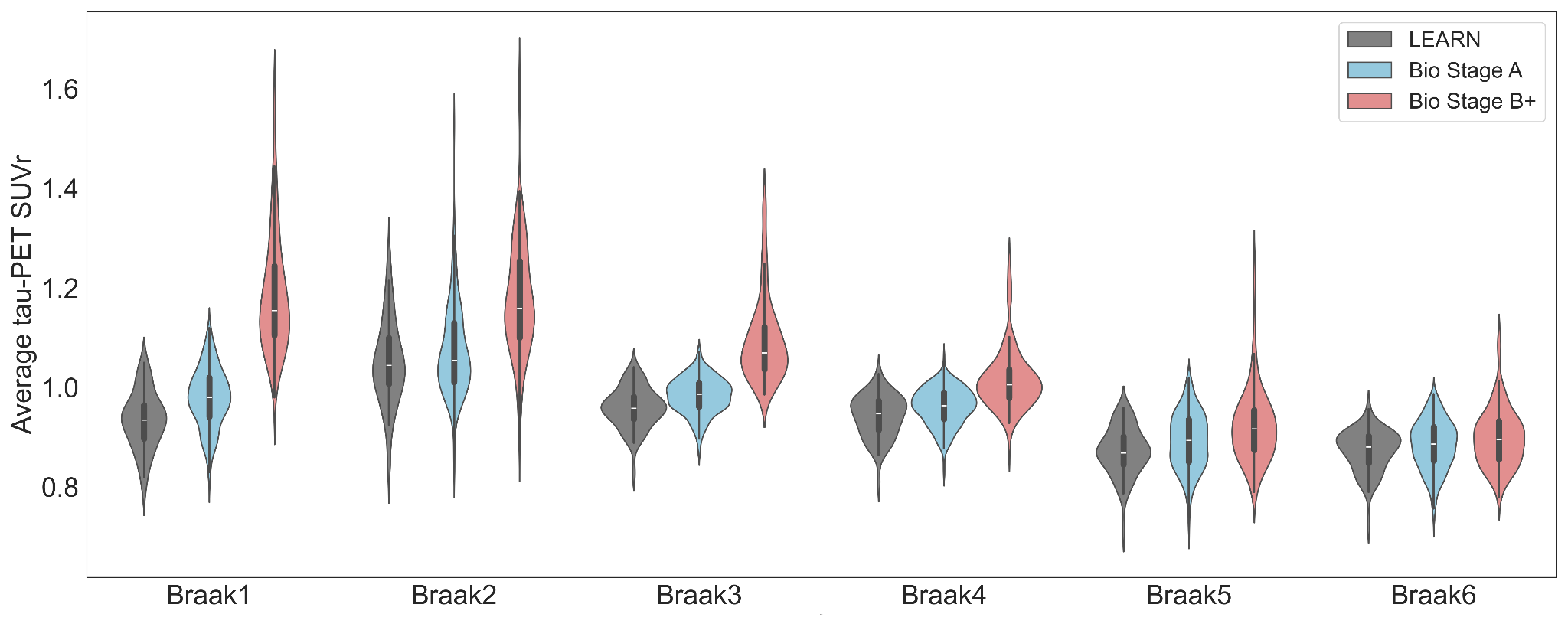
**

**Supplemental Results 5. Braak ROI SUVrs across Biological Stages.** Grey, blue, and red represent the distributions for the average Braak SUVr for the LEARN (amyloid-negative), Biological Stage A, and Biological Stage B+ groups, respectively. Each Braak SUVr is an average of the regional SUVrs of the regions that make up that specific Braak meta-ROI [(3)](https://paperpile.com/c/TJ3uCs/gbxhJ)). Given that the splitting of the sample into Bio Stage A and B+ was based on MTL tau-PET alone, we aimed to evaluate whether participants with temporal-sparing tau-PET patterns were included in the Biological Stage A group. Participants with relative temporal sparing tau-PET patterns have been described previously in A4 [(1)](https://paperpile.com/c/TJ3uCs/U8oAV). While there are Biological Stage B+ participants with elevated average SUVr in Braak 3-6 regions, there is no indication that this is the case for the Biological Stage A group. The individual ROIs that make up each Braak meta-ROI are as follows. Braak 1: entorhinal; Braak 2: hippocampus; Braak 3: parahippocampal, fusiform, lingual, amygdala; Braak 4: inferior and middle temporal, temporal pole, thalamus, insula, and cingulate; Braak 5: frontal, parietal, and occipital cortex, superior temporal, precuneus, nucleus accumbens, caudate, and putamen; Braak 6: precentral, postcentral, paracentral, cuneus, and pericalcarine. Abbreviations: ROI=region of interest; SUVr=standardized uptake value ratio; MTL=medial temporal lobe.


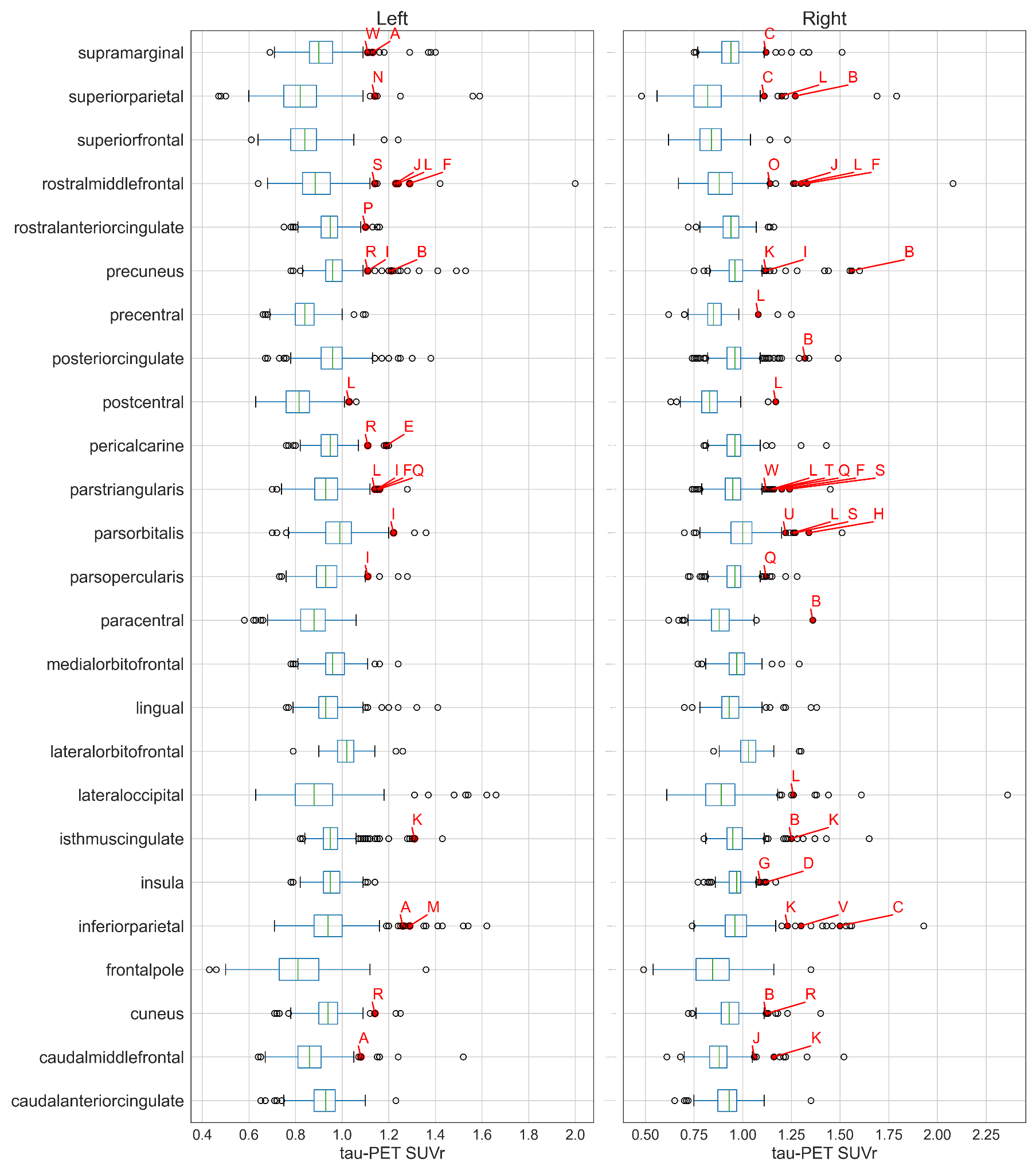


**Supplemental Results 6. Regional tau-PET SUVr distributions.** In this figure, we show the individual SUVr distributions for cortical regions of interest across the LEARN and A4 cohort. This procedure was performed to see if there were any participants within our Biological Stage A that may have temporal-sparing focally-elevated tau-PET. We identified participants that had at least one outlier regional SUVr (outlier was defined as a regional SUVr greater than 1.5*IQR above the third quartile). We identified 23 such participants in Biological Stage A. Their outlier SUVrs are labeled with a red dot in the distributions and the individual participants are labeled with a letter (letters A-W). We removed these 23 participants from our analyses and performed the longitudinal LME analyses as a sensitivity analysis (our main manuscript uses the originally defined 222 Biological Stage A participants to keep consistent with the updated criteria that focuses on the MTL tau-PET for classification into Stage A versus B). The model estimates are in Supplemental Table 2 and the pattern of results from sensitivity analyses is discussed above in Supplemental Results 4.

**References**

1. [Young CB, Winer JR, Younes K, Cody KA, Betthauser TJ, Johnson SC, et al. Divergent cortical tau positron emission tomography patterns among patients with preclinical Alzheimer disease. JAMA Neurol. 2022 Jun 1;79(6):592–603.](http://paperpile.com/b/TJ3uCs/U8oAV)

2. [Jack CR Jr, Andrews JS, Beach TG, Buracchio T, Dunn B, Graf A, et al. Revised criteria for diagnosis and staging of Alzheimer’s disease: Alzheimer's Association Workgroup. Alzheimers Dement. 2024 Aug 1;20(8):5143–69.](http://paperpile.com/b/TJ3uCs/AQUXC)

3. [Schöll M, Lockhart SN, Schonhaut DR, O’Neil JP, Janabi M, Ossenkoppele R, et al. PET imaging of tau deposition in the aging human brain. Neuron. 2016 Mar 2;89(5):971–82.](http://paperpile.com/b/TJ3uCs/gbxhJ)
